# Incidence of Implant Removal in Surgically Treated Patella Fractures

**DOI:** 10.7759/cureus.25377

**Published:** 2022-05-26

**Authors:** Shivanshu Mittal, Vivek k David, Vikas Sharma

**Affiliations:** 1 Orthopedic Surgery, Manipal Tata Medical College, Jamshedpur, IND; 2 Orthopedic Surgery, Tata Main Hospital, Jamshedpur, IND; 3 Orthopedics, Gouri Devi Institute of Medical Sciences & Hospital, West Bengal, IND; 4 Orthopedics, Tata Main Hospital, Jamshedpur, IND; 5 Biometrics, Cliantha Research, Ahmedabad, IND; 6 Biostatistics, Tata Main Hospital, Jamshedpur, IND

**Keywords:** cannulated screws, kirschner wire, tension band, implant removal, patella fracture

## Abstract

Context: Patella fracture is frequently encountered in day to day practice of every orthopedic surgeon, yet conventional modalities of fixation that are associated with a high incidence of implant removal continue to be standard practice.

Aim: To estimate the incidence of implant removal in surgically managed patella fractures.

Settings and design: Retrospective study.

Materials and methods: Data of surgically managed patella fractures in our institution from January 2017 to December 2019 was collected from the hospital management system and analyzed retrospectively. Statistical Package for Social Sciences (SPSS) version 23 (IBM Corp., Armonk, NY, USA) was used for the analysis of data. Nominal and categorical variables were analyzed using Chi-square, and continuous data were expressed as mean and standard deviation. A p-value ≤0.05 was considered significant.

Results: The study group had a total of 106 patients, with 74 males and 32 females and an average age of 47.67 ± 17.85 years. Most commonly the fractures occurred following an accidental fall and road traffic accident. A comminuted pattern was most common. The mean length of stay was 7.34 ± 4.21 days. Osteosynthesis with Kirschner wire and tension band wires was done in 83 cases. The average follow-up was 13 months (range, three to 48 months). Twenty-two patients (one in five) required a second surgery for implant removal and the rate was higher in patients more than 60 years of age, females, and comminuted fractures. Cases managed with cannulated cancellous screws did not require any removal. The average timing of removal was 14 months (range, seven to 28 months). Relief of symptoms following removal was observed in 86% of cases.

Conclusion: Conventional fixation techniques offer good union rates but at a cost of a high incidence of removal. Multi-centric trials are required to compare the rates of removal of conventional methods with new techniques being adopted.

## Introduction

Fractures of the patella, the largest sesamoid bone of the body, account for about 1% of all skeletal system injuries [1-4]. The Patella fractures after a direct or indirect force that acts on it. Direct forces, such as anterior blow, fall on the knee, and dashboard injury often results in >50% undisplaced/minimally displaced, comminuted, or stellate fracture pattern with severe chondral damage and intact retinaculum [1,2]. Indirect forces act when the knee is suddenly flexed on contracted quadriceps. When this force exceeds the strength of bone, the patella fractures, and the retinaculum tears leading to a transverse displaced pattern [2]. Conservative treatment is offered to patients with minimal displacement of the fracture and when there is no significant incongruity of the joint surface [5]. The treatment modalities have evolved and are now more focused on preserving the knee extensor mechanisms. Open reduction and osteosynthesis using Kirschner and cerclage wires remain the gold standard in treating patella fractures where there is articular incongruity or step of more than 2mm to 3mm [4-7]. Most of the cases are managed on the tension band principle. Yet, some comminuted fractures require additional stabilization in addition to tension band construct in the form of additional Kirschner wires (K-wires), cerclage wires/cables, and interfragmentary screws [8]. However, the management remains challenging due to the subcutaneous, intra-articular location of the patella and may require a second surgery for the removal of hardware as the implants become prominent when the swelling subsides, and the patient develops soft tissue irritation, pain, or restriction of motion [3,9]. Many novel techniques are thus being tried such as transosseous sutures of different make, mesh plates, arthroscopy assisted percutaneous pinning/screws, titanium-nickel shape memory patella concentrator, patella locking star plate (Arthrex®, Arthrex Inc., Naples, FL, USA) [4,5,9-11]. The current study was performed in a tertiary care institution and sought to estimate the incidence of implant rate removal following open reduction and osteosynthesis of patella fractures and identify any factors which have a positive correlation with increased removal rates.

## Materials and methods

Between 1 January 2017 and 31 December 2019, 123 patients aged 13 to 91 years were operated for patella fractures in our institution, out of which 17 were excluded. The data acquisition is from the hospital management system after clearance from the institutional ethics committee. Patients who succumbed due to any reason (n=5) and those who did not follow up and could not be contacted telephonically (n=12) were excluded from the study. The records were reviewed for the age and sex of the patient, mechanism of injury, closed or open injury, side of injury, associated fractures, method of surgical intervention, length of stay, postoperative complications, duration of follow-up, telephone numbers, and if removal was done. The X-rays were reviewed to assess the pattern of fracture, type of fixation, if the union was achieved, broken implant, or implant migration. Patients who did not visit for regular follow-ups were reached telephonically to gather required information. Reason and timing of implant removal, relief of symptoms after removal were documented. The data collected was entered in Microsoft Excel (Microsoft Corp., Redmond, Washington, USA) and Statistical Package for the Social Sciences (SPSS) version 23 (IBM Corp., Armonk, NY, USA) was used for filtration and statistical analysis. Nominal and categorical variables were recorded as frequency and percentages and analyzed using Chi-square where applicable. Continuous data were expressed as mean and standard deviation. A p-value of ≤0.05 was considered significant.

## Results

The study group had a total of 106 patients, with 74 males and 32 females with an average age of 47.6 ± 17.85 years (Table 1). The modes of injury are described in Table 2. The majority of patients sustained an injury following accidental slip and fall or road traffic accidents. One patient sustained a sports-related injury and had an associated patellar tendon injury. Fourteen patients had open injuries. All the open injury patterns were operated on in emergency on the same day. Twenty-seven patients had other associated fractures, the majority of these (n=21) had fractures in the femur (distal femur or shaft) following a road traffic accident (n=23). The fracture patterns are detailed in Table 3, with the majority being comminuted and two-part transverse. The mean length of stay in the hospital was 7.34 ± 4.21 days. All the surgeries were performed using a midline longitudinal skin incision. The most common mode of fixation was the routinely done K-wire and tension band wiring (n=83). The various hardware configurations done are entered in Table 4. All the cases were followed up for a mean duration of 13 months (range, three to 48 months).

**Table 1:** Demographics and data SD = Standard deviation

S.No	Variable	Result
1.	Gender	
a)	Males	74 (69.81%)
b)	Females	32 (30.19%)
2.	Laterality	
a)	Left	58
b)	Right	48
3.	Mean age ± SD (in years)	47.67 ± 17.85
4.	Mean Length of stay ± SD (in days)	7.34 ± 4.21
5.	Mean follow-up ± SD (in months)	13.17 ± 9.43
a)	No implant-related issues	12.32 ± 9.92
b)	Underwent removal	16.45 ± 6.42

**Table 2:** Mode of Injury RTA = Road Traffic Accident

S.no	Mode of injury	Number of cases
1.	Accidental fall	54
2.	RTA	46
3.	Fall from stairs	2
4.	Fall from height	3
5.	Sports-related	1
	Total	106

**Table 3:** Fracture pattern

Fracture pattern	Cases
Two-part transverse	38
Comminuted	51
Two-part vertical	4
Superior pole	1
Inferior pole	12
Total	106

**Table 4:** Configurations of surgical hardware in patients operated K-wire = Kirschner wire, TBW = Tension band wiring, CC screws = Cannulated cancellous screws

S.no	Type of fixation	Number of cases
1.	K-wires+TBW	83
2.	K-wires+TBW+cerclage	8
3.	K-wires+TBW+CC screws	2
4.	Cerclage	8
5.	CC screws	4
6.	CC screws +TBW+cerclage	1
	Total	106

Twenty-two patients underwent implant removal, of which 10 had localized pain due to skin impingement causing restriction of knee range of motion, eight had prominence with irritation, two had pain with broken implants, one developed superficial infection, and one patient had only prominence. All the cases underwent implant removal after fracture union was confirmed. Fifteen patients who underwent removal were less than 60 years of age and the others were 60 years and above. Their average time of removal was 14 months (range, seven to 28 months) and follow-up was 16 months (range, eight to 36 months). Removal was significantly more in comminuted fracture (p-value=0.0343) and high in females (Table 5). The elderly above 60 years of age experienced increased rates of removal (23.3%) in an average span of 11 months (range, seven to 16 months) after surgery when compared to those less than 60 years of age where 19.7% underwent removal after a mean duration of 16 months (range, eight to 28 months). None of the cases with cannulated screws underwent removal.

**Table 5:** Implant removal data

Variables	Total cases	<60years	>60 years	Males	Females	Comminuted fracture	Other fracture patterns
Number	22	15	7	13	9	15	7
Average (%)	20.5	19.7	23.3	17.5	28.1	29.4	12.7

Relief of symptoms after implant removal was observed in 19 patients (86%). One patient who developed infection had persistent pain, and two patients continued to have restricted range of motion but also had associated distal femur and proximal tibia fractures. The rehabilitation course followed in most patients depended upon fracture configuration. The fracture patterns without comminution were taught knee range of motion to 90 degrees or tolerable limits of pain, non-weight bearing mobilization, and isometric quadriceps strengthening exercises on the second postoperative day. A gradual increase in knee flexion was started after suture removal at two weeks to regain preoperative range of motion within the next four weeks. Partial weight-bearing started with crutches at four weeks and allowed full weight-bearing after confirming union radiologically and gait training. Comminuted patterns were immobilized with a splint for three weeks and taught non-weight-bearing mobilization and isometric quadriceps strengthening. Knee range of motion till 90 degrees from three to six weeks with toe-touch, beyond 90 degrees along with partial weight-bearing after six weeks, and allowed full weight-bearing after confirming union or as tolerated.

## Discussion

Patella plays a pivotal role in maintaining the integrity of the knee extensor mechanism acting as a fulcrum of the lever of the quadriceps. Thus, any disruption needs intervention to help the patient regain knee extension and independent mobilization [11,12]. A conservative approach is often followed only if there is no articular step, the fracture is undisplaced or the patient is not fit for surgery. A comminuted pattern (48%, n=51) is found more often than other patterns in clinical studies [8]. A midline longitudinal or transverse skin incision is used for exposing the fracture site for osteosynthesis, although transverse is not recommended by the Association of Osteosynthesis (AO) [13]. The most commonly used tension band wiring converts tensile forces into compressive forces at the fracture site when the knee is flexed through the range of motion [14,15]. This allows early mobilization to avoid arthrofibrosis of the knee, and quadriceps wasting and is beneficial for articular cartilage nutrition [4,15,16]. As the fragmentation increases, supplemental fixation in the form of cerclage or K-wires, screws, cables/pins are added, based on the surgeon’s preference. Augmentation is also done when the bone is osteoporosed and there are increased chances of cut-through. Since more focus is now being made on preserving the extensor mechanism, none of the cases in the current series underwent partial or total patellectomy. However, this delays the rehabilitation protocol as complex three-dimensional forces, including bending, tension and torsion act upon the patella-femoral surface as the fracture heals and early mobilization can lead to early fixation failures, requiring revision surgery for fracture disruption or broken/migrated implants [8,17,18].

Implant removal is done when the patient complains or demands one. There are no set protocols guiding the timing of routine removal. Removal surgeries are well associated with risks and complications including anesthetic risks, chances of surgical site infection and delayed wound healing, a period of immobilization, poor cosmetics due to scarring, and the economic burden to the society. Also, the second surgery may not provide relief of symptoms, as was observed in three patients in our study. Greenberg et al. suggested that removal gives significant pain relief but doesn’t significantly improve the functional limitations [19]. We observed an overall rate of removal to be 20.5% (one in five cases) which is lower when compared to other published literature reporting removal rates at 17% to 70% [3,8,12,17,19-21]. The left or right side had no relation to removal rates (equivocal). Though low, cases managed with cannulated screws in our series did not require removal. Literature reports support evidence of low removal rates with screws. The low profile of screw heads making them less prominent, and relatively low chances of migration over K-wires explains the outcome [3,4,17]. The rates were more in the elderly aged 60 and above (23.3%). Also, the elderly group underwent removal before one year of index surgery (average=11 months). This can be explained by the increased chances of early cut-through due to osteoporosed bone, loosening, and migration and since the skin is fragile, implants become easily prominent. Literature suggests conflicting observations and advocates fewer chances of removal in the elderly due to low functional demand and attributed high functional activity and increased forces acting on extensor mechanism leading to increased chances of removal in the young [3,12,15,20]. The comminuted pattern had significantly higher rates of removal (29.4%) relative to other fracture configurations (12.7%). This is an expected measurement as the amount of hardware used is more, and so are the chances of migration and loosening. The results described in the current study are per existing literature. To the best of our knowledge, there is no published data from the Indian subcontinent which has calculated the removal rates.

Surgeons should be wary of these estimates, predictable risk factors such as old age, and comminuted patterns, and explain to their patients the chances of a second surgery. Also, do take informed consent before proceeding with the surgery. Limitations of this study include a small number of fixation failure cases for statistical analysis amongst various subsets of patients, retrospective analysis, and lack of functional scorings. As the surgeries were performed by a team of different orthopedic consultants in a single institution, techniques and skills differ. Also, all the patients did not follow a single rehabilitation course as some surgeons take up a conservative approach while some are more aggressive.

## Conclusions

The outcome of surgical fixation of patella fractures by conventional techniques offers good union rates to date. Yet, it doesn’t solve the implant-related issues which is a concern in this frequently encountered case in day to day practice of orthopedic surgeons. Although the number of cases managed with cannulated cancellous screws was relatively low, they did not require any removal which has been observed in other literature as well. The elderly, females, and comminuted patterns observed high removal rates. Further research and literature are required to observe and compare the rates of removal in newer techniques that are being adopted.
